# One‐Step Generation of a Drug‐Releasing Hydrogel Microarray‐On‐A‐Chip for Large‐Scale Sequential Drug Combination Screening

**DOI:** 10.1002/advs.201801380

**Published:** 2018-11-20

**Authors:** Seo Woo Song, Su Deok Kim, Dong Yoon Oh, Yongju Lee, Amos Chungwon Lee, Yunjin Jeong, Hyung Jong Bae, Daewon Lee, Sumin Lee, Jiyun Kim, Sunghoon Kwon

**Affiliations:** ^1^ Department of Electrical and Computer Engineering Seoul National University Seoul 08826 South Korea; ^2^ Institutes of Entrepreneurial BioConvergence Seoul National University Seoul 08826 South Korea; ^3^ Interdisciplinary Program in Bioengineering Seoul National University Seoul 08826 South Korea; ^4^ Department of Mechanical Science and Engineering University of Illinois at Urbana‐Champaign Urbana IL 61801 USA; ^5^ Nano Systems Institute Seoul National University Seoul 08826 South Korea; ^6^ School of Materials Science and Engineering Ulsan National Institute of Science and Technology Ulsan 44919 South Korea

**Keywords:** drug‐laden hydrogel, encoded microparticle, high‐throughput screening, self‐assembly, sequential combination

## Abstract

Large‐scale screening of sequential drug combinations, wherein the dynamic rewiring of intracellular pathways leads to promising therapeutic effects and improvements in quality of life, is essential for personalized medicine to ensure realistic cost and time requirements and less sample consumption. However, the large‐scale screening requires expensive and complicated liquid handling systems for automation and therefore lowers the accessibility to clinicians or biologists, limiting the full potential of sequential drug combinations in clinical applications and academic investigations. Here, a miniaturized platform for high‐throughput combinatorial drug screening that is “pipetting‐free” and scalable for the screening of sequential drug combinations is presented. The platform uses parallel and bottom‐up formation of a heterogeneous drug‐releasing hydrogel microarray by self‐assembly of drug‐laden hydrogel microparticles. This approach eliminates the need for liquid handling systems and time‐consuming operation in high‐throughput large‐scale screening. In addition, the serial replacement of the drug‐releasing microarray‐on‐a‐chip facilitates different drug exchange in each and every microwell in a simple and highly parallel manner, supporting scalable implementation of multistep combinatorial screening. The proposed strategy can be applied to various forms of combinatorial drug screening with limited amounts of samples and resources, which will broaden the use of the large‐scale screening for precision medicine.

## 1 Introduction

Treating diseases with multiple drugs leads to more complex and elaborate cellular pathway regulation.^1^ Thus, finding effective drug combinations has been of interest for a long time, and the discovered drug combinations have been applied to cure patients with resistant cancers that were difficult to treat with single‐drug therapy.^2^, ^3^ However, the concurrent administration of multiple drugs increases dose exposure in patients at a specific moment and, therefore, has significant potential to result in side effects.^4^, ^5^ To address this limitation, sequential treatment with multiple drugs has received much attention.^6^, ^7^ Recently, several studies have reported the sequence‐dependency of some drug combinations, which is more powerful than concurrent combinations.^8^, ^9^, ^10^, ^11^ The underlying principle is the dynamic rewiring of intracellular pathways in which the pretreated drug makes the cell status vulnerable to the post‐treatment drug.^12^, ^13^ If such an effective sequential combination can be found for each patient, thus resulting in personalized medicine, it can provide not only a promising therapeutic effect but also help to improve the quality of life by reducing the drug dose given to the patient.^4^, ^5^

Finding effective drug combinations for a patient generally requires unbiased large‐scale screening.^14^, ^15^ However, the number of cells obtained from a patient is usually limited; thus, only a few combinations can be tested for cell samples by using conventional high‐throughput screening (HTS) platforms (i.e., 96‐ or 384‐well‐plate‐based platforms).^16^, ^17^ To overcome this limitation, various types of HTS platforms have evolved to reduce the reaction volume, thereby decreasing the consumption of valuable cells and reagents.^18^, ^19^, ^20^, ^21^ These platforms only need nanoliter or picoliter amounts of reagents and a few hundred cells per reaction, thus making large‐scale drug screening with patient‐derived samples possible.^16^, ^17^ However, as the screening platform becomes miniaturized and the scale of screening expands, increasingly sophisticated and expensive liquid handling systems are required for managing the large number of drug candidates to be tested.^22^ Because the majority of hospitals and laboratories worldwide have difficulty securing proper infrastructure and extra funding to operate such a high‐cost liquid handling system, it is challenging for them to utilize HTS for clinical applications and academic studies.^16^, ^17^

**Figure 1:**
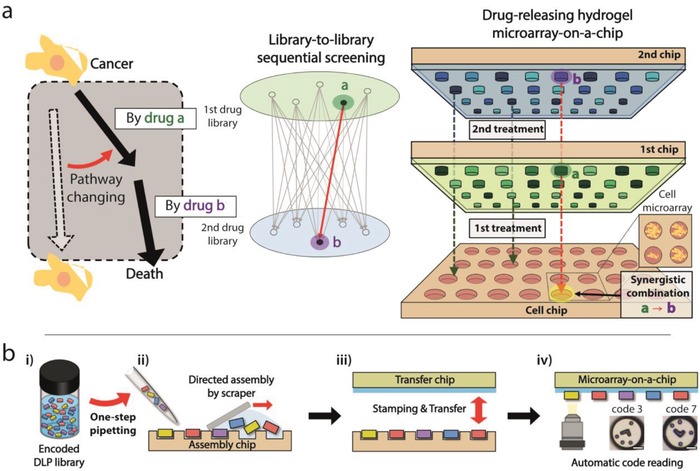
Large‐scale sequential combination screening by serial replacement of a drug‐releasing hydrogel microarray‐on‐a‐chip. a) Principle of rewiring cellular pathways by effective sequential drug treatment, and schematic illustration of multistep drug treatment in a highly parallel manner to find an efficient sequential drug pair. The drug‐releasing hydrogel microarray‐on‐a‐chip is combined with the cell chip to facilitate the multiplexed bioassay. Simple exchange of the microarray‐on‐a‐chip enables sequential delivery of heterogeneous drug combinations to corresponding microwells in a parallel manner. b) Schematic illustration of how the large‐scale, drug‐releasing hydrogel microarray‐on‐a‐chip is constructed by fully manual operation with a reduced workload. (i) Encoded drug‐laden microparticles (DLPs) are moved from the library to the assembly chip by one‐step pipetting. (ii) Encoded DLPs are simply assembled into microwells on the assembly chip by scraper‐assisted directed assembly. (iii) Array of DLPs is transferred to the transfer chip by stamping. (iv) Codes of encoded DLP are decoded automatically to identify which drug treatment was applied to each microwell.

This study aimed to provide an affordable screening tool for performing sequential drug screening with a reduced workload and sample requirement. To achieve this goal, we developed a miniaturized “pipetting‐free” HTS platform for sequential combinatorial screening with high scalability (**Figure** **1**). There are multiple advantages to our platform. First, the cost of HTS significantly decreases because this platform eliminates the need for an expensive liquid handling system, which accounts for the majority of the cost of implementing HTS. We developed a bottom‐up formation method of heterogeneous drug microarray that was formed by the self‐assembly of encoded drug‐laden microparticles (DLPs) on a microwell array (Figure 1b). This scalable method requires just one‐step pipetting to provide a large‐scale drug‐releasing microarray‐on‐a‐chip. Several previous studies have attempted to achieve pipetting‐free HTS platforms, but these techniques were limited to preparing a homogeneous microarray (i.e., cell microarray).^23^, ^24^, ^25^ Second, the simple and robust implementation of multistep (or sequential) drug treatment is enabled by serially replacing drug‐releasing microarrays combined with a cell microarray (Figure 1a). Our technique is easily scalable for large‐scale, sequential combinatorial screening in particular because only the *n*‐step pipetting operation, instead of the exponentially increasing liquid handling operations, is sufficient for *n*‐step drug combination screening. We demonstrated a sequential combinatorial screening of a targeted inhibitor followed by genotoxin against triple‐negative breast cancer (TNBC), which is known to be an especially highly resistant form of breast cancer. As a result, we found that erlotinib followed by mitoxantrone showed the most promising effect among tested combinations and investigated their synergism from the dose–response matrix. To our best knowledge, this pair had not been discovered prior to our study. We believe that our proposed technique can narrow the gap between HTS and individual laboratories with limited resources, thereby expanding the application of HTS in various fields. We envision that our platform can contribute especially to finding efficient drug combinations for each patient and accelerate the era of personalized medicine.

## 2 Results

### 2.1 Preparation of Drug‐Releasing Hydrogel Microarray‐on‐a‐Chip

The preparation of a drug‐releasing hydrogel microarray‐on‐a‐chip is conducted by “partipetting,” a word that represents a combination of the words “particles” and “pipetting.”^26^, ^27^ This word indicates the delivery of heterogeneous DLPs with single pipetting and the directed self‐assembly of DLPs into microwells of an “assembly chip” (Figure 1b‐i,ii). With the partipetting method, thousands of heterogeneous drugs can be delivered simultaneously; thus, one‐step pipetting is required for one chip. Subsequently, assembled DLPs are transferred to a “transfer chip,” which has a thin PDMS layer on a polystyrene chip, thereby constructing a drug‐releasing hydrogel microarray‐on‐a‐chip (Figure 1b‐iii). Because DLPs are randomly assembled during partipetting, a code‐reading process is implemented to identify which drug is delivered to which microwell (Figure 1b‐iv). A DLP code indicates the drug with which a microparticle is impregnated.

### 2.2 Validation of Partipetting Platform for Multiplexed Cell‐Based Assay

Encoded hydrogel microparticles, which are used as drug carriers, were fabricated by photolithography with a photocurable polymer (polyethylene glycol diacrylate, *M*_n_ = 700). These particles have a diameter of 138 µm and a thickness of 38 µm. Polymerized hydrogels were washed with ethanol two times to remove the uncured monomer and were easily collected into the test tube or well plate by using a blade (Figure S1a, Supporting Information). No cytotoxicity caused by hydrogel microparticles was found (Figure S1b, Supporting Information). A library of drug‐laden hydrogel microparticles could be prepared in a simple and highly parallel manner, which is described as follows (Figure S1c, Supporting Information). The drug solution was added into the microwells with prefabricated microparticles, and the solvent was removed by freeze‐drying. Because commercial drug libraries are generally supplied in a 96‐ or 384‐well plate format and microparticles can also be supplied in a well plate format, the DLPs‐supplier or the end‐user only needs to transfer the drug solution and freeze‐dry the mixture. After they were completely dried, the microparticles in each microwell were collected into inert silicone oil to construct a DLP library. Here, silicone oil prevents cross‐contamination between different DLPs^26^ and functions to deliver liquid during the partipetting process. Most of the silicone oil that remains after the assembly process was removed, and then the array of DLPs was transferred to the transfer chip. Thus, there was no residual silicone oil to disturb the release of the drug or change the culture condition.

**Figure 2:**
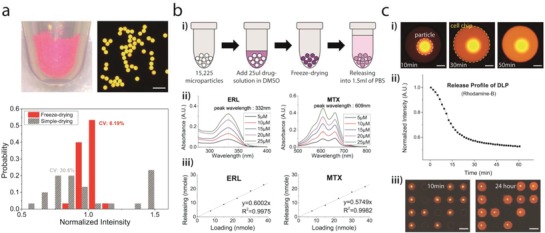
Drug delivery into the microwell using a microparticle. a) The loading uniformity of the freeze‐drying‐based loading method. Rhodamine‐B was used as a model substance, and the fluorescence intensity was measured to estimate the loading amount of molecules. The loading uniformity was significantly improved compared to a conventional drug loading method (see also Figure S2, Supporting Information). Scale bar: 500 µm. b) Measuring drug releasing ratio. (i) The releasing ratio of each drug was measured from a bulk‐scale releasing experiment. After drug loading by freeze‐drying, 1.5 mL of PBS was added to the DLPs. The concentration of released solution was measured after the overnight releasing process. (ii, iii) The UV–vis absorbance spectrum was used to measure the concentration of released solution. For all drugs, released amounts of drugs were linearly proportional to the initial loading amount (see also Figure S3 and Table S1, Supporting Information). Data of erlotinib and mitoxantrone are shown as representatives. c) Drug releasing into microwells and isolation of each microwell during incubation. (i) Image of a microwell with Rhodamine‐B releasing microparticle. Scale bar: 150 µm. (ii) Fluorescence intensity from a microparticle from time‐lapse imaging shows that the releasing process was completed within 30–60 min. (iii) The isolation of each microwell was maintained over the 24 h incubation time. Scale bar: 1 mm.

As opposed to our previous study on the partipetting platform,^26^ we adopted the freeze‐drying‐based drug‐loading method in this study to attain a uniform and high amount of drug loading regardless of the drug or solvent type.^28^ With rhodamine‐B as a model substance, we investigated the loading uniformity by measuring the fluorescence intensity of the microparticles (**Figure** **2**a, and Figure S2, Supporting Information). The loading uniformity from the previous method (gray) was 30.6% and was improved 6.19% by freeze‐drying (red). The number of drug molecules that could be delivered to a microwell with a single microparticle could be estimated from a bulk‐scale loading–releasing experiment (Figure 2b, Figure S3, Supporting Information, and the Experimental Section). After drug loading, 1.5 mL phosphate‐buffered saline (PBS) solution was added to the DLPs, and then the mixture was shaken on a mixing block overnight, which was enough time for complete release (Figure 2b‐i). We set the volume of the PBS solution to make the particle number (15225) to the releasing volume (1.5 mL) ratio equivalent to the ratio of a single particle to the volume of one microwell on the cell chip (100 nL) (Table S2, Supporting Information). The concentration of the released solution was measured using an ultraviolet–visible absorbance spectrum (Figure 2b‐ii, and Figure S3a,b, Supporting Information). As a result, we validated that the released amount of drug was linearly proportional to the initial loading amount (Figure 2b‐iii, and Figure S3c, Supporting Information). Therefore, once the releasing ratios of the drugs were examined, we could easily modulate the target concentration by controlling the loading amount of the drugs (Table S1, Supporting Information).

For the cell‐based assay, cells were seeded on the “cell chip” that has microwells with a diameter of 600 µm and a depth of 350 µm. The gravitational settling method or the sealing film‐assisted seeding method was used for cell plating (Figure S4, Supporting Information). The sealing film‐assisted seeding method was developed to reduce the number of cells required for the assay, which is applicable to rare cell screening, such as seeding stem cells or patient‐derived cells (Figure S4b, Supporting Information). Cell seeding on 1600 microwells was available only with 320 µL, which can fill only three microwells on the 96‐well plate. Here, the total volume of microwells is 160 µL, but two times more volume of cell suspension was used to fill all of the microwells without bubbles. This seeding method could be completed within 10 s, and the seeding uniformity was within 10% of the coefficient variation. After preparing the cell chip and drug‐releasing hydrogel microarray‐on‐a‐chip, both chips were combined in a face‐to‐face manner to conduct multiplexed cell‐based bioassays. Sequential treatment assays were available by simply replacing the microarray‐on‐a‐chip after incubation.

Drug molecules in hydrogel microparticles start to diffuse out after a microparticle touches a cell culture medium. During the incubation, each microwell should be completely isolated to prevent cross‐contamination. This diffusion was validated with rhodamine‐B (Figure 2c). The impregnated molecules in the microparticle were gradually released into the surrounding solution, and the releasing process was completed within ≈30–60 min (Figure 2c‐i,ii). We also ensured that the isolation of each microwell was maintained perfectly for at least 1 d (Figure 2c‐iii). If the releasing speed was too fast, cross‐contamination between microwells would occur during the chip assembly process, which requires ≈5 s. In contrast, if the releasing speed were too slow to finish the releasing process within the drug incubation time, it would be difficult to obtain accurate assay results. Considering that the incubation time for an anticancer drug assay is usually more than 10 h, the releasing speed of our DLPs is acceptable for use in drug screening. Conversely, the sealing of microwells may inhibit gas exchange during the incubation period, which could affect the cell status. To understand this potential impact, we investigated the influence of sealing the cells in terms of cell morphology and viability, and there was neither a morphological change nor a decrease in the survival rate due to sealing (Figure S5, Supporting Information).

### 2.3 Decoding Process and Distribution of Sequential Combinatorial Codes

**Figure 3:**
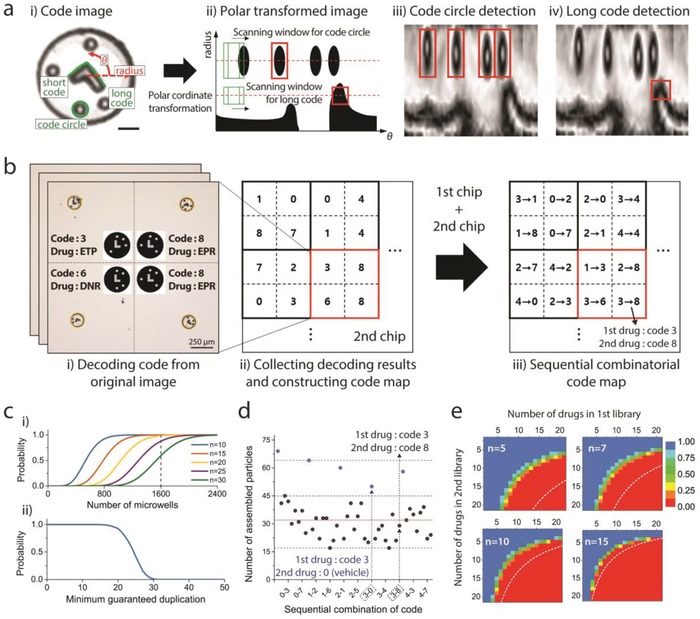
The automatic decoding and statistical analysis of code distributions. a) Decoding algorithm. (i) “Long code,” “short code,” and “code circles” are components for identifying the rotation, inversion, and code number of microparticles, respectively. Scale bar: 30 µm. (ii)–(iv) Particle image is transformed into polar coordinates, and the locations of each code component are recognized by a machine‐learning‐based algorithm. b) Construction of sequential combinatorial code map. Decoding results from all images are collected, and a sequential combinatorial code map is generated. c) Probability about the minimum duplication number. (i) Probabilities that a “specific” combination has a duplicate greater than *n* according to the number of microwells. (ii) Probability that “all” combinations will be found over a certain number of duplicates in 1600 microwells. d) Example of combinatorial code distribution. First and second drug libraries contain four and eight kinds of drugs, respectively. Total 45 combinations (including single or no drug treatment, represented by blue dots) were found from 1600 microwells. Red line (*n* = 32) is the expected value except for the cases in which no particle is applied in the second treatment (blue dots). e) Available library size for sequential combination screening in a single chip (1600 microwells). Color represents the probability of partipetting to guarantee that all combinations have duplications greater than *n*. White dashed lines are available library size for conventional pipetting technique.

To identify which sequential combination of drugs was treated on each microwell from randomly assembled DLPs, we utilized encoded microparticles and developed automatic code‐reading software (**Figure** **3**a,b). Three code elements were used to construct the graphical barcode (Figure 3a‐i). The “long code” and “short code” were utilized to analyze the rotation angle and the upside‐down inversion of a microparticle, respectively. After aligning the direction of a microparticle using long code and short code, the code of a microparticle is determined according to the location of “code circles” (Figure S6a,b, Supporting Information). Without long code and short code, it is not possible to know whether the particle is rotated or flipped, and therefore, different codes that have the same relative position between code circles cannot be distinguished (Figure S6c, Supporting Information). For the reading process, the detected particle image was transformed into polar coordinates first, and the positions of the code circles and the long code were recognized in the scanning window traversing the scanning line (Figure 3a‐ii). To avoid a decoding error caused by the truncation of coding components, polar transformation was conducted for 405° (360° and additional 45°) along the theta axis. A short code can be detected easily by comparing the intensity of the area at −90° and +90° from the long code position (θ). Finally, the code of the particle was determined according to the relative location between the coding components. By using neural‐network‐based image recognition and subsequent error‐correction procedure, more than 95% of the particles were successfully decoded. The error under the 5% includes the broken particles whose code cannot be decoded. Finally, a sequential combinatorial code map was constructed by combining the code maps of the first and second chips (Figure 3b).

Because DLPs are randomly assembled into microwells, an important evaluation point regarding the feasibility of our platform is whether sufficient duplication numbers are guaranteed for all combinations. We carried out statistical analysis to answer this question. The assembly yield was 80.79%, and a binomial distribution was used to calculate the theoretical probabilities of combinations of code distributions. First, we examined the probabilities with 45 sequential combinations, which were used for the screening that is demonstrated in the final section of this paper. The probabilities that a “specific” combination has a minimum duplication number (*n*) according to the number of microwells are represented (Figure 3c‐i). However, to guarantee the minimum duplication numbers for “all” combinations, a different probability model is required. When microparticles are assembled in a limited number of microwells once the duplication number of a specific combination is determined, the probabilities of the remaining combinations should be calculated under the number of remaining microwells (see also the Supporting Information). Such a probability of guaranteeing the minimum duplication number for all combinations can be modeled numerically with the Monte Carlo method (Figure 3c‐ii). The probability was almost 1.0 until the minimum guaranteed duplication number was 15, and one example of an actual distribution of sequential combinatorial codes showed results that matched the simulation result (Figure 3d). Next, we examined how many sequential drug combinations could be screened in a single chip (1600 microwells) to ensure a certain number of duplications (Figure 3e). Red‐to‐blue colors represent the probability that partipetting guarantees a duplication number greater than *n* (in each inset). Each white dashed line represents the maximum library size that is available in the situation that all drugs are conveyed to the decided position of a microwell with a conventional liquid handling technique, which is not dependent on random assembly. There is a considerable difference in the number of screenable drugs between our platform and that of the conventional platform for small *n* (i.e., *n* = 3). This is an inevitable drawback of our platform in implementing pipetting‐free HTS based on the random assembly of microparticles. However, if the desired number of duplications is large enough (i.e., *n* = 15), the difference between the library sizes of screenable drugs for partipetting and conventional technologies is negligible. Since it is common to obtain a large number of duplicates to increase the accuracy on a miniaturized HTS platform that uses a small cell number, we consider that this shortage of our platform is not serious enough to diminish the advantage of using our platform, which can dramatically reduce the workload for large‐scale bioassays. For a limited number of cells, such as patient‐derived primary cells, the proposed platform can test many more drug candidates compared with that of conventional screening platforms (Table S3, Supporting Information).

### 2.4 Easy‐to‐Use Platform and Polystyrene Chip Fabrication

**Figure 4:**
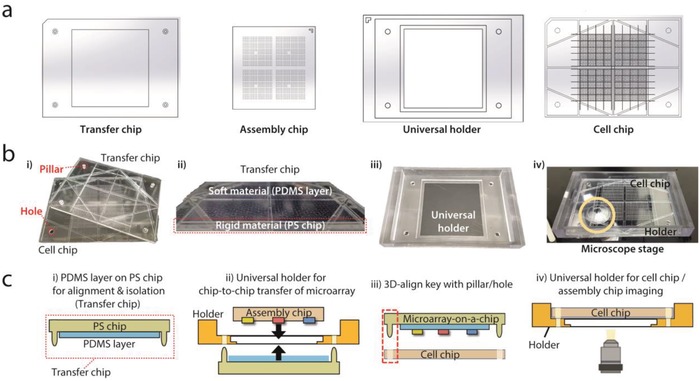
A polystyrene (PS) chip system fabricated by injection molding and a multipurpose holder for easy alignment. a) PS chip system with 1600 microwells and universal holder for alignment. b) Images of chip components. (i) Cell chip and transfer chip (hydrogel microarray‐on‐a‐chip) are combined with the help of holes on the cell chip and pillars on the transfer chip. (ii) A two‐layer system composed of a PDMS sheet on the PS chip was applied to the transfer chip. Rigid PS body with pillars enables the simple alignment of a hydrogel microarray on a target cell chip, and an elastic PDMS sheet facilitates complete sealing between the cell chip and transfer chip (microarray‐on‐chip). (iii, iv) Universal holder was designed to assist alignment between the assembly chip and transfer chip and imaging on the microscope at a predefined position. c) Schematic illustration of experimental procedure. (i, ii) DLP array on the assembly chip is transferred to the transfer chip with the aid of the universal holder. (iii) The hydrogel microarray‐on‐chip and cell chip are combined through a 3D alignment key composed of pillars/holes. (iv) Universal holder helps to locate the cell chip and assembly chip in a predefined position on the microscope.

Soft lithography using PDMS traditional manufacturing technologies for microwells is useful for prototyping, but it is too expensive and slow for mass production.^29^ Additionally, it is hard to conduct precise chip‐to‐chip aligning processes between two combined microwell arrays due to the flexible and stretchable characteristics of PDMS. Thus, we fabricated our platform with polystyrene (PS) chips by injection molding, which is an easily scalable strategy for rigid‐plastic components with a 3D alignment key with pillar and hole structures to help perform sequential replacements easily (**Figure** **4**a,b). However, PS chips can have a surface roughness on the micrometer‐scale, and this roughness of the surface of PS chips is likely to create a slight gap between two combined chips, which may cause cross‐contamination between adjacent microwells. This cross‐contamination could potentially lead to serious problems in microwell‐based technology, since each microwell should perform a separated and isolated reaction. To ensure isolation between adjacent microwells to prevent a cross‐contamination problem, we devised a new chip called the transfer chip, which has a two‐layered structure, with an elastic PDMS sheet on top of the rigid PS chip (Figure 4b‐ii,c‐i). If the rigid PS chip were used solely, it would be hard to prevent cross‐contamination, because it is difficult to fabricate a plastic chip with uniform roughness in a micrometer‐scale. This soft and elastic PDMS layer can help prevent the problem by filling the gap between two plastic chips after two plastic chips were combined. For the stamping of DLPs array, the attraction force between the PDMS and microparticles made of PEGDA (*M*_n_ = 700) was strong enough to achieve a transfer yield of more than 99% and maintain adhesion after 1 d of incubation. There is no need for further surface treatment on the PDMS layer, and the stamping of the DLPs array could be conducted with only finger pressure. By carefully controlling some parameters, such as the depth and diameter of each microwell and the distance between adjacent microwells, we manufactured a set of large‐scale PS chips that can perform 1600 parallel reactions within a 52 mm × 52 mm area.

A schematic illustration of how each chip and the universal holder is used in each step is shown in the experimental order (Figure 4c). Assembled DLPs on the assembly chip are transferred to the transfer chip with the help of a multipurpose plastic holder (named a “universal holder”) for exact alignment. Then, large‐scaled imaging for positional code mapping is performed. The imaging process is implemented on the universal holder, which is designed to be the same size and is compatible with the microscopic stage for conventional 96‐well plates; thus, all the microparticles are located at preset coordinates. Subsequently, combining the transfer chip (microarray‐on‐a‐chip) and the cell chip induces drug release. In this process, pillars on the transfer chip and holes in the cell chip facilitate an exact alignment. After incubation, whole microwells on the cell chip with drug‐treated cells are imaged on the top of the universal holder, thereby collecting viability information on the whole chip.

### 2.5 Multiplexed Sequential Drug Combination Assay

**Figure 5:**
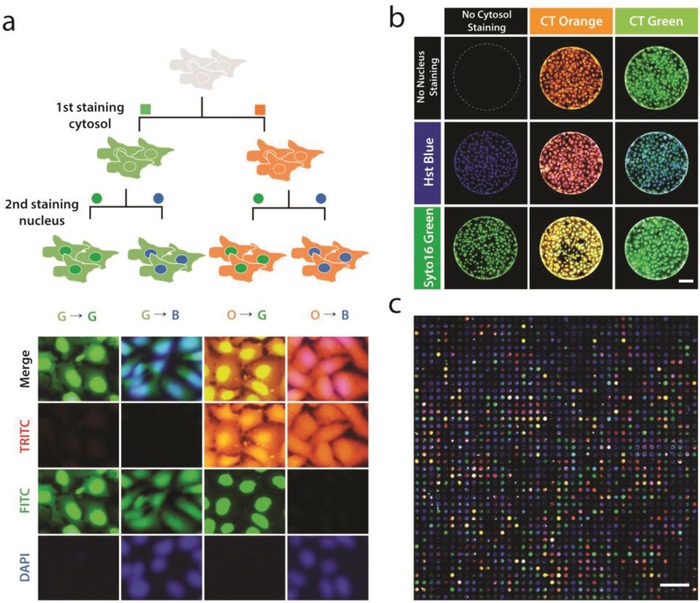
Sequential combinatorial cell staining. a) Schematic illustration of the staining process, and magnified images of stained cells. Cell Tracker (CT) green and orange were used for the first staining, and Syto 16 (green) and Hoechst (Hst) 33342 (blue) were used for the second staining. Scale bar: 50 µm. b) All possible combinations of sequential staining. In total, nine combinations were found on the whole chip, including the case in which staining dyes were not treated. Scale bar: 150 µm. c) Staining image of the whole chip with 1600 microwells. Scale bar: 5 mm.

We previously validated the availability of the partipetting platform to screen concurrent combinatorial drugs by controlling the design of the placement of microwell arrays on the assembly chip.^26^ However, the previous version of our platform could support only a single incubation step. In this work, we demonstrated a sequential combination assay with the concept of exchanging a drug‐releasing hydrogel microarray‐on‐a‐chip. This becomes available with the advantages of the 3D‐align key on rigid plastic chips. For the proof‐of‐concept, a sequential cell staining assay was first demonstrated by using cytosol staining with green and orange CellTracker dyes followed by nucleus staining with blue Hoechst 33342 and green SYTO 16 nucleic acid staining dyes (**Figure** **5**). Two drug‐releasing hydrogel microarrays‐on‐a‐chip were combined sequentially on one cell chip. Hydrogel microarray‐releasing cytosol‐staining dyes and nucleus‐staining dyes were placed on the first and second chips, respectively. It was confirmed that the fluorescence images of the cells were clearly distinguished depending on the combination of staining dyes treated sequentially (Figure 5a). In total, nine combinations were possible by using staining dyes, and we could find all of them from the images of the microwells on the cell chip (Figure 5b). For a large‐scale experiment, a sequential combination staining experiment was conducted on a large‐scale chip with 1600 microwells (Figure 5c). From these results, we validated that the sequential delivery of drugs to each isolated microwell is available in a scalable manner by exchanging a hydrogel microarray‐on‐a‐chip.

**Figure 6:**
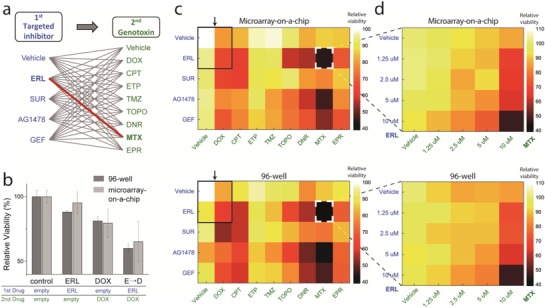
Large‐scale sequential drug combination assay against BT‐20 using drug‐releasing hydrogel microarray‐on‐a‐chip. a) The list of screened sequential combinations. Library‐to‐library screening with targeted inhibitor treatment followed by genotoxin was implemented. b) Synergistic effect of sequential combinations. The results of erlotinib and doxorubicin are shown as representative examples. c) Sequential combination screening results from our platform using a microarray‐on‐a‐chip and a conventional 96‐well‐based technique. Erlotinib followed by mitoxantrone was revealed as the most synergistic sequential pair (highlighted in white box) in the screened combination library. Black boxes indicate the data represented in (b). d) Dose–response matrix screening for erlotinib followed by mitoxantrone. In all heat maps, the color of each spot represents the relative viability based on a negative control without any treatment (vehicle → vehicle). The color map on the right shows the percentage value of relative viability corresponding to the color. All viability data from the proposed platform and 96‐well plate were within ±15% of each other.

Finally, we applied the proposed platform to conduct large‐scale screening of the anticancer efficacy of sequential drug combinations (EGF receptor inhibitor followed by a DNA damaging agent) against the triple‐negative BT‐20 cell line (**Figure** **6**). TNBC has remained an aggressive cancer without effective single‐targeted therapies, thus trials of treating patients with combinatorial drugs are being administered widely.^30^ The list of screened drug combinations is shown in Figure 6a and in Table S1 (Supporting Information). In the experiments, the final concentration of all drugs released from DLP was adjusted to 10 × 10^−6^ m (Table S1, Supporting Information), and each sequential drug was incubated for 12 h with inhibitory drugs and 10 h with the DNA damaging agents (time‐table shown in Figure S7, Supporting Information). For all combinations, the experimental results from 96‐well plates and our platform had similar drug efficacies (Figure 6c). The cytotoxicity of single‐drug therapies and the sequential treatment from one combination, erlotinib and doxorubicin (indicated as a black box in Figure 6c), are represented in the bar graph (Figure 6b). Although our platform has a higher CV value (≈15%) than that of the 96‐well platform (≈3%), it is a trade‐off caused by the small reaction volume and fewer cell numbers. From the screening results, erlotinib followed by mitoxantrone was revealed to be the most effective sequential pair among the total 45 combinations. The survival rate of cells treated with this pair was 44.46% in experiments using our platform and 40.63% in 96‐well plate experiments. To evaluate the synergism of the combination, a dose–response matrix was also obtained using our platform (Figure 6d). All of the results from the proposed platform were within ±15% of those obtained by using a conventional approach, which proved the feasibility of our platform for sequential drug combination assays.

## 3 Discussion

In this study, we described a large‐scale screening platform to find effective sequential drug combinations. The delivery of encoded drug‐laden microparticles using one‐step pipetting and the self‐assembly of these microparticles to an array of microwells can replace thousands of pipetting operations. For a multistep drug incubation, only a simple exchange of the hydrogel microarray‐on‐a‐chip is required instead of repeating thousands of pipetting operations for every treatment step. Furthermore, since our platform supports the screening of concurrent combinatorial drugs, this technique can apply to the various forms of combination screening. Such a significant decrease in the workload would give hospitals and laboratories with limited resources the opportunity to perform large‐scale, multistep bioassays at an affordable cost and within a reasonable timeframe. Regarding the required number of samples, only 200 cells per microwell were needed, and the uniform seeding of 1600 microwells was possible without a robotic pipette machine through the sealing film‐assisted seeding method. To make this platform usable by other researchers, we designed an easy‐to‐use platform by introducing 3D pillar/hole structures and a multipurpose holder for easy alignment.

It has not been long since researchers discovered that the effects of sequential combinations can be attributed to the dynamics of signaling pathways. Therefore, there are many opportunities for new achievements, as our biological understanding of disease deepens. We envision that personalized screening results from sequential combinations will help determine drug schedules for each patient.^14^, ^17^ In addition, our platform for multistep combinatorial screening can potentially be applied in other research fields, such as the differentiation and reprogramming of various cell types,^24^, ^31^, ^32^, ^33^, ^34^ the performance of drug screening using cells transduced with various viral vectors,^35^, ^36^ or the creation of personalized drug scheduling using patient‐derived cell lines. We expect that multistep, large‐scale screening using partipetting will be more accessible to researchers in a wide range of fields, thereby broadening the applications of high‐throughput, sequence‐dependent combinatorial bioassays.

## 4 Experimental Section

*Encoded Microparticle Fabrication*: Encoded hydrogel microparticles were fabricated via UV photolithography (OmniCure S1500, Excelitas Technology Corp.) with poly(ethylene glycol)‐diacrylate (PEGDA, *M*_n_ = 700; Sigma‐Aldrich) and 5 wt% photoinitiator (2‐hydroxy‐2‐methylpropiophenone 97%, Sigma‐Aldrich). To generate different codes for each hydrogel microparticle, different masks (MicroTech, South Korea) with the capacity to generate 15 225 microparticles at once were used. All of the fabricated hydrogel particles were first collected in an ethyl alcohol (EtOH) solution. To prevent the photoinitiator residue of uncured resin from damaging cells, the washing steps with fresh EtOH solution were repeated two times, and then dried. Here, the photomask for making microparticles of a diameter 150 µm was used, but the fabricated microparticle slightly shrinks to 138 µm diameter after drying. However, all of the coding components shrunk at the same rate while maintaining their shape; thus, there was no problem in reading the code.

*Chip Fabrication*: All of the plastic chips, including the assembly chip, transfer chip, and cell chip, were manufactured by plastic injection molding using an injection mold (Woojin Selex Co., Ltd., South Korea). An aluminum mold was made using a CNC milling machine (Hwacheon Technology, South Korea). All of the chips, which were made of polystyrene (GPPS, LGChem, South Korea), contained 1600 microwells. The microwells of the cell chip had a diameter of 0.6 mm and a well‐to‐well distance of 1.5 mm. The microwells on the assembly chip had a diameter of 160 µm and a well‐to‐well distance of 1.5 mm (the same distance as the wells on the cell chip).

*Preparation of DLPs and Measuring Drug‐Releasing Ratio*: Erlotinib hydrochloride and gefitinib (free base) were purchased from LC Laboratories, and all of the other chemical drugs were purchased from Sigma Aldrich. The drug concentration was measured with an ultraviolet–visible spectrophotometer (UV‐1800, Shimadzu). First, the peak wavelength of absorbance spectrum was measured for each drug (Figure 2b‐ii, and Figure S3a, Supporting Information). Subsequently, a reference curve for the relationship between the drug concentration and corresponding absorbance peak was obtained by using samples with a known concentration (Figure S3b, Supporting Information). Then, the concentrations of the unknown samples can be calculated from the reference curve (Figure S3c, Supporting Information). To evaluate the drug‐releasing ratio of each drug, the following procedure was conducted. First, drug solution (dissolved in 25 µL dimethyl sulfoxide (DMSO)) at a known concentration was added to 15 225 microparticles, which were fabricated from a single mask. After freeze‐drying, PBS solution (1.5 mL) was added to drug‐laden microparticles, and then the mixture was shaken on a mixing block (MB‐102, BIOER) overnight, which was enough time for complete release. Next, the concentration of the released solution was measured. The particle number (15 225) to the releasing volume (1.5 mL) ratio was equivalent to the ratio of a single particle to the volume of one microwell (100 nL) (Table S2, Supporting Information). It was tried to eliminate the differences in the releasing ratios that might occur depending on the particle numbers and volume ratios. Five data points for each drug were collected (see also Figure 2, Figure S3 and Table S1, Supporting Information).

*Cell Culture*: BT‐20 human mammary gland/breast carcinoma cells (ATCC) and U2OS human osteosarcoma cells (KCLB, Korean Cell Line Bank) were cultured in Eagle's minimum essential medium (EMEM) and McCoy's 5A culture medium, respectively. Both media were supplemented with 1% penicillin‐streptomycin and 10% fetal bovine serum (at 37 °C under 5% CO_2_ and 95% atmospheric air). Cultured cells were trypsinized to detach them from the culture flask by using 0.25% trypsin and 0.13% EDTA in phosphate‐buffered saline. The cells were centrifuged and dispersed in the cell culture medium. All of the plastic chips were sterilized with EtOH sonication, oven dried at 60 °C, and given oxygen plasma treatment for 4 min.

*Sequential Cell Staining*: To demonstrate heterogeneous sequential cell staining, green and orange CellTracker (0.25 × 10^−3^ m, 25 µL, Invitrogen) were loaded into the microparticles for cytosol staining. For the second staining, Hoechst 33342 (blue, 0.05 × 10^−3^ m, 25 µL) and SYTO 16 (green, 0.05 × 10^−3^ m, 25 µL, Invitrogen) were loaded into the microparticles for nucleus staining. The incubation time for each staining step was 4 h.

*Cell Viability Assay*: For the cell viability assay, Calcein AM (Thermo Fisher Scientific) was used to stain the live cells. ≈1 mg mL^−1^ Calcein AM solution was diluted in serum‐free EMEM culture media at a 1:1000 ratio, and the cells were incubated with the solution for 30 min. After PBS washing, fluorescence images of the microwells on a cell chip were obtained by using a microscope setup with a motorized stage (Nikon Digital Sight DS‐Ri1, Nikon C‐LHGFI HG LAMP). A fluorescein isothiocyanate (FITC) channel filter (excitation 490 nm, emission 525 nm) with an exposure time of 200 ms for a 2× objective lens or 60 ms for 4× lens were used for image acquisition. The viability was decided by relative pixel intensity from the fluorescence image of a microwell area compared with that of nondrug‐treated microwells (the control). For the average viability of each drug combination, 10% of the upper and lower value data were excluded as outliers.

## Conflict of Interest

The authors declare no conflict of interest.

## Supporting information

Supplementary
