# Supplementary material for: One‐Step Generation of a Drug‐Releasing Hydrogel Microarray‐On‐A‐Chip for Large‐Scale Sequential Drug Combination Screening
Source: Adv Sci (Weinh). 2018 Nov 20;6(3):1801380. doi: 10.1002/advs.201801380 (PMC6364496; doi:10.1002/advs.201801380)
Supplement: Supplementary file 1 — Supplementary [file ADVS-6-1801380-s001.pdf]

Copyright WILEY-VCH Verlag GmbH & Co. KGaA, 69469 Weinheim, Germany, 2018.

ADVANCED SCIENCE
Open Access

Supporting Information

for *Adv. Sci.,* DOI: 10.1002/advs.201801380

One-Step Generation of a Drug-Releasing Hydrogel Microarray-On-A-Chip for Large-Scale Sequential Drug Combination Screening

Seo Woo Song, Su Deok Kim, Dong Yoon Oh, Yongju Lee, Amos Chungwon Lee, Yunjin Jeong, Hyung Jong Bae, Daewon Lee, Sumin Lee, Jiyun Kim, and Sunghoon Kwon**

Copyright WILEY-VCH Verlag GmbH & Co. KGaA, 69469 Weinheim, Germany, 2016.

Supporting Information

One-Step Generation of a Drug-Releasing Hydrogel Microarray-on-a-Chip for Large-Scale Sequential Drug Combination Screening

Seo Woo Song, Su Deok Kim, Dong Yoon Oh, Yongju Lee, Amos Chungwon Lee, Yunjin Jeong, Hyung Jong Bae, Daewon Lee, Sumin Lee, Jiyun Kim*, and Sunghoon Kwon*

Supporting Method

Statistical analysis of code distribution with binomial distribution and Monte Carlo simulation

Partipetting process utilizes the random assembly of microparticles into the microwells, thus combination code distribution is depended on assembly yield. We experimentally obtained 80.79% of assembly yield (CV: 7.63% from 47 experimental set). For the following probability modeling process, the probability (p) that certain microwell is filled with microparticle was set to 80% (p = 0.8).

If we have N microwells in an assembly chip, the probability that n microwells are filled with particles follows a binomial distribution. Thus, the probability that the number of occupied microwells (X) is over than n is like below (cumulative binomial probability).

$$P(X \geq n) = \sum_{i=n}^{N} \binom{N}{i} p^i (1-p)^{N-i}$$

Because we used various microparticles with different codes, the probability (p') that microparticles with certain code is assembled into microwell is p divided by number of drugs in the library (L).

$$p' = p/L$$

For the sequential combination from the libraries with L_1 and L_2 drugs, probability ($P_{specific\ combi}$) that specific code combination have duplication number (X) over than n becomes

$$P_{specific\ combi}(X \geq n) = \sum_{i=n}^{N} \binom{N}{i} p''^{i} (1-p'')^{N-i}$$

where, $p'' = \frac{p}{L_1} \times \frac{p}{L_2}$

This probability is represented in Figure 3c-ii in the manuscript.

However, as written in the manuscript, number of duplications for combinatorial codes are not determined independently. If the first combinatorial code has duplication of X_1, then the probability that 2nd and 3rd combinatorial code have duplications over than n follows

$$P(X_2 \geq n) = \sum_{i=n}^{N-X_1} \binom{N-X_1}{i} p''^{i} (1-p'')^{N-X_1-i}$$

$$P(X_3 \geq n) = \sum_{i=n}^{N-X_1-X_2} \binom{N-X_1-X_2}{i} p''^{i} (1-p'')^{N-X_1-X_2-i}$$

With the inductive logic, numbers of duplications of remaining combinatorial codes are determined. We calculated this probability with Monte Carlo simulation with 5,000 trials for each experimental setting, and the results are presented in Figures 3c-i and 3e.

Supporting Figures

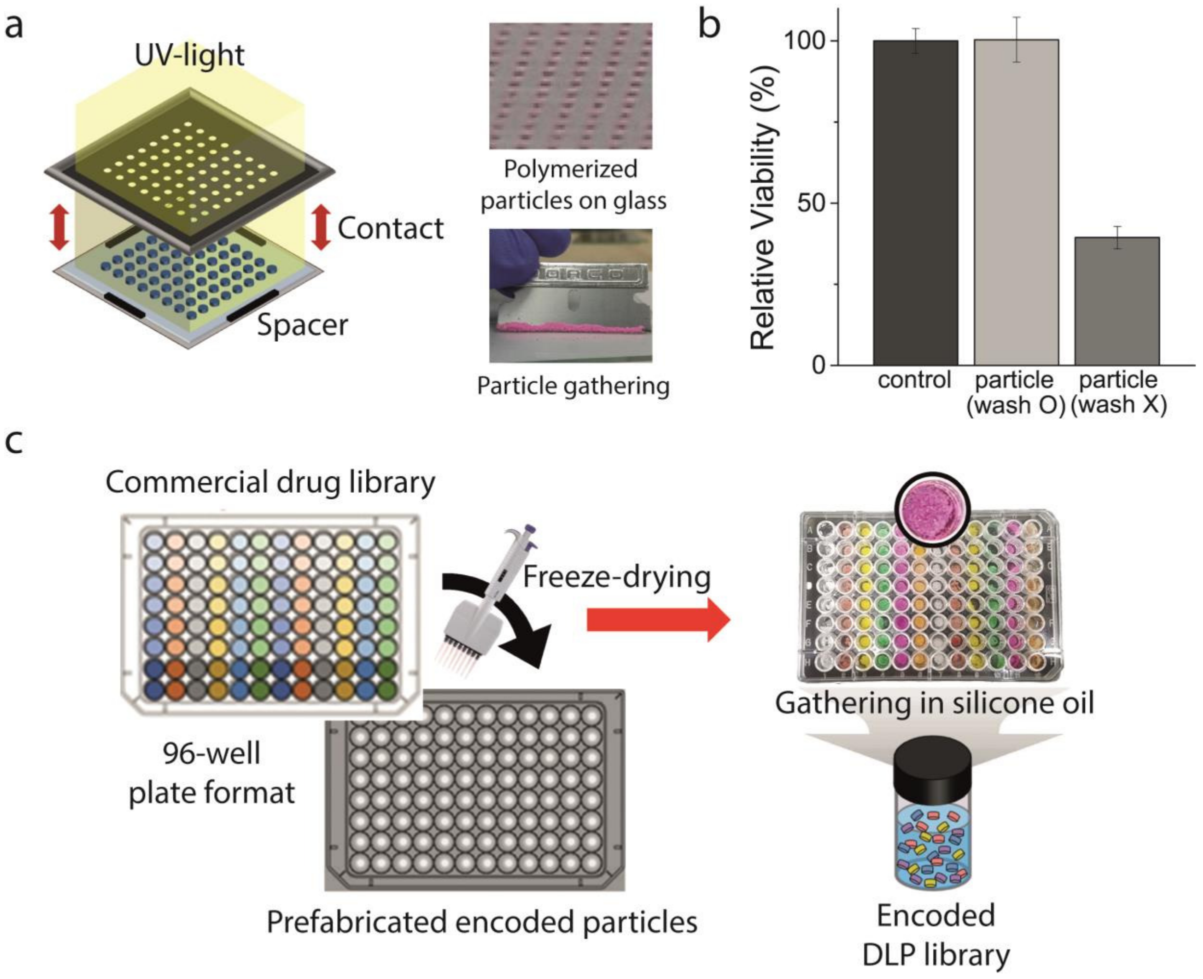

Figure S1. Constructing library of encoded drug-laden particles (DLP library) and its biocompatibility. a) Encoded hydrogel microparticles were fabricated by mask photolithography. 45 μm of spacer was applied to adjust the height of microparticles. 15,225 microparticles were polymerized simultaneously on a single mask, and polymerized microparticles were gathered using a blade. b) Biocompatibility of hydrogel microparticle as a drug carrier was investigated. Although microparticles with uncured monomer showed toxic effect to the cells, two-times washed microparticles with ethanol did not have cytotoxicity. c) Encoded DLP library can be prepared in a simple and highly parallel manner. Commercial drug library is generally supplied in a 96- or 384-well plate format, and encoded hydrogel microparticles are also available to be supplied in a well plate format. The end-user only needs to transfer drug solution using multi-pipette and freeze-dry the mixture.

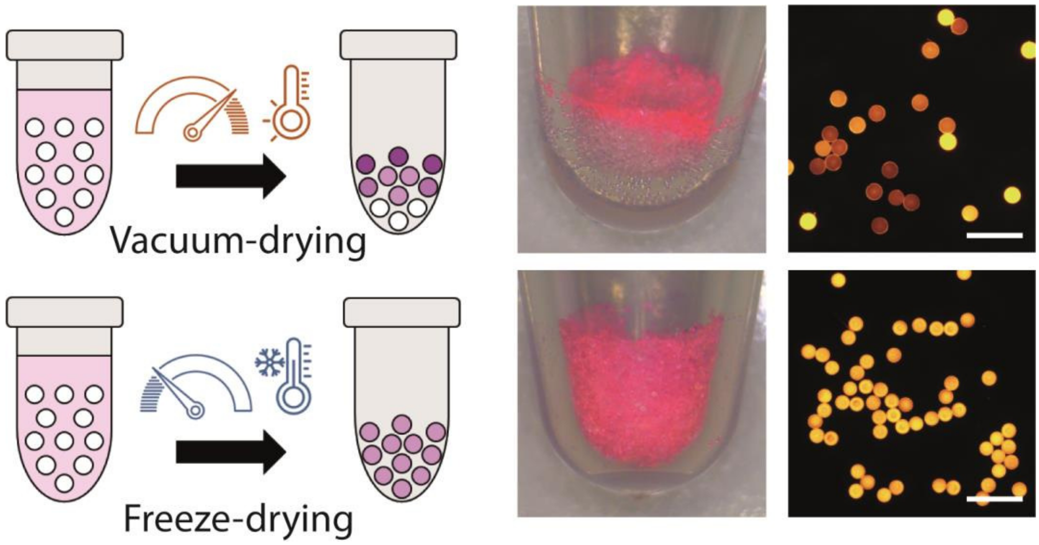

Figure S2. Drug loading into the prefabricated encoded hydrogel microparticles using freeze-drying. Drug loading based on solvent removal from the mixture of microparticles and drug solution can attain high amount of drug loading regardless of drug type or properties of microparticles. However, capillary flow during the solvent removal process cause non-uniform drug loading when vacuum-drying is used for drug loading. Because no capillary flow occurs during sublimation (freeze-drying), uniform loading is available. As shown in Figure 2a, uniformity of loading by solvent evaporation and freeze-drying were investigated with a model substance Rhodamine-B. Coefficient of variation (CV) values are 30.6% and 6.19% for simple vacumm drying and freeze-drying, respectively. Scale bar: 500 µm.

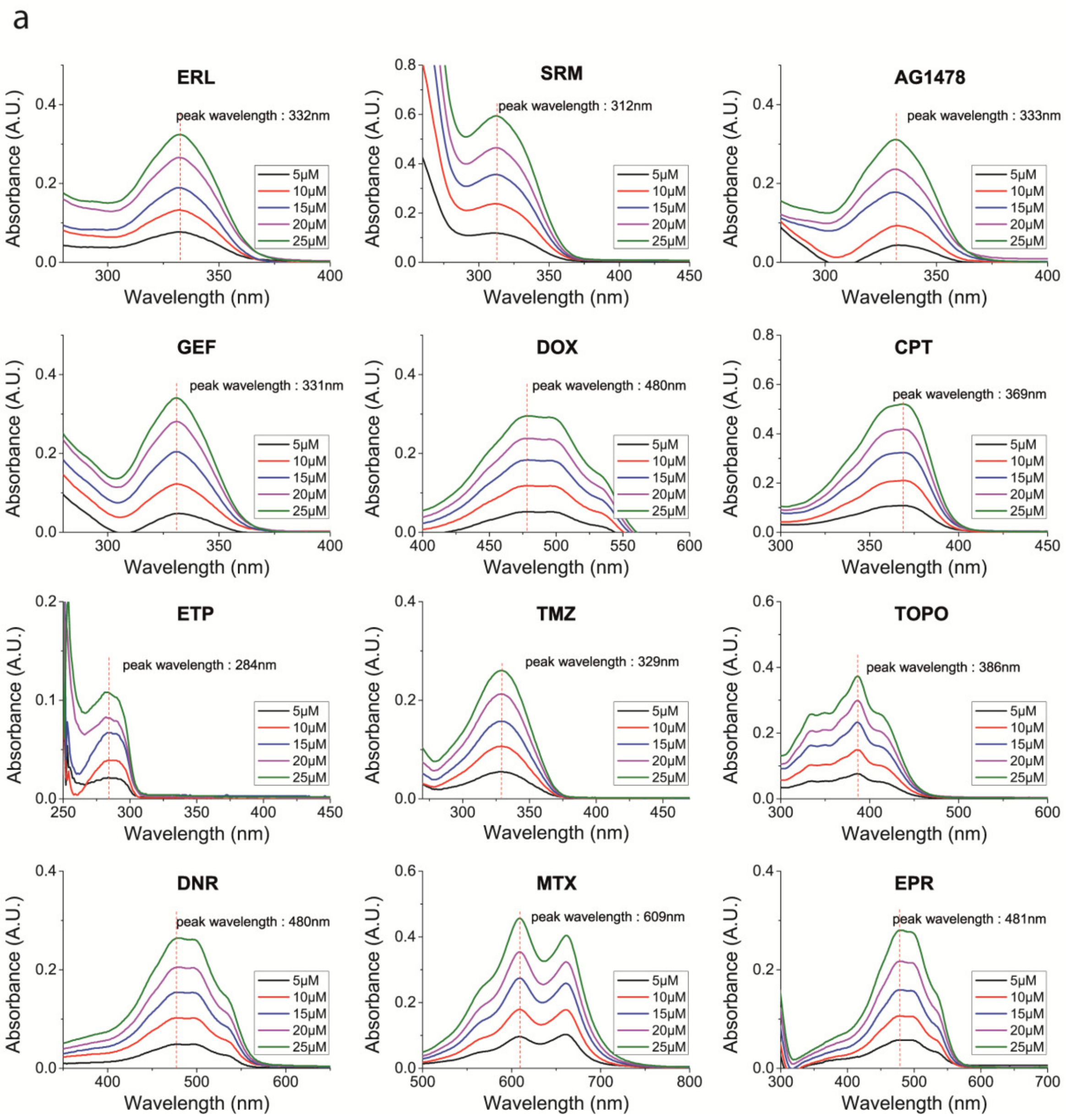
a
ERL
peak wavelength : 332nm
SRM
peak wavelength : 312nm
AG1478
peak wavelength : 333nm
GEF
peak wavelength : 331nm
DOX
peak wavelength : 480nm
CPT
peak wavelength : 369nm
ETP
peak wavelength : 284nm
TMZ
peak wavelength : 329nm
TOPO
peak wavelength : 386nm
DNR
peak wavelength : 480nm
MTX
peak wavelength : 609nm
EPR
peak wavelength : 481nm
5μM
10μM
15μM
20μM
25μM
Absorbance (A.U.)
Wavelength (nm)

b

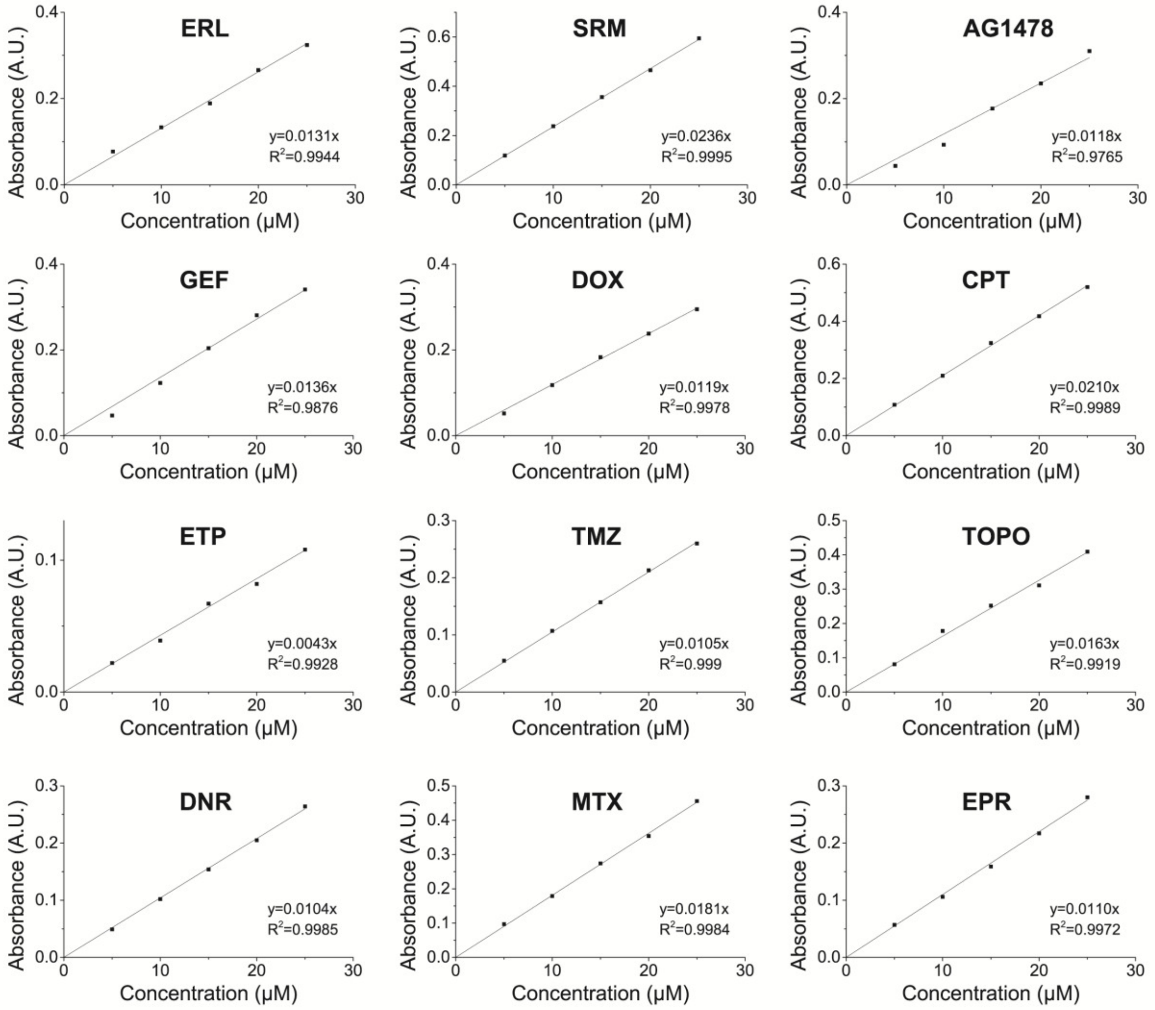
ERL
y=0.0131x
R^2=0.9944
SRM
y=0.0236x
R^2=0.9995
AG1478
y=0.0118x
R^2=0.9765
GEF
y=0.0136x
R^2=0.9876
DOX
y=0.0119x
R^2=0.9978
CPT
y=0.0210x
R^2=0.9989
ETP
y=0.0043x
R^2=0.9928
TMZ
y=0.0105x
R^2=0.999
TOPO
y=0.0163x
R^2=0.9919
DNR
y=0.0104x
R^2=0.9985
MTX
y=0.0181x
R^2=0.9984
EPR
y=0.0110x
R^2=0.9972
Absorbance (A.U.)
Concentration (μM)

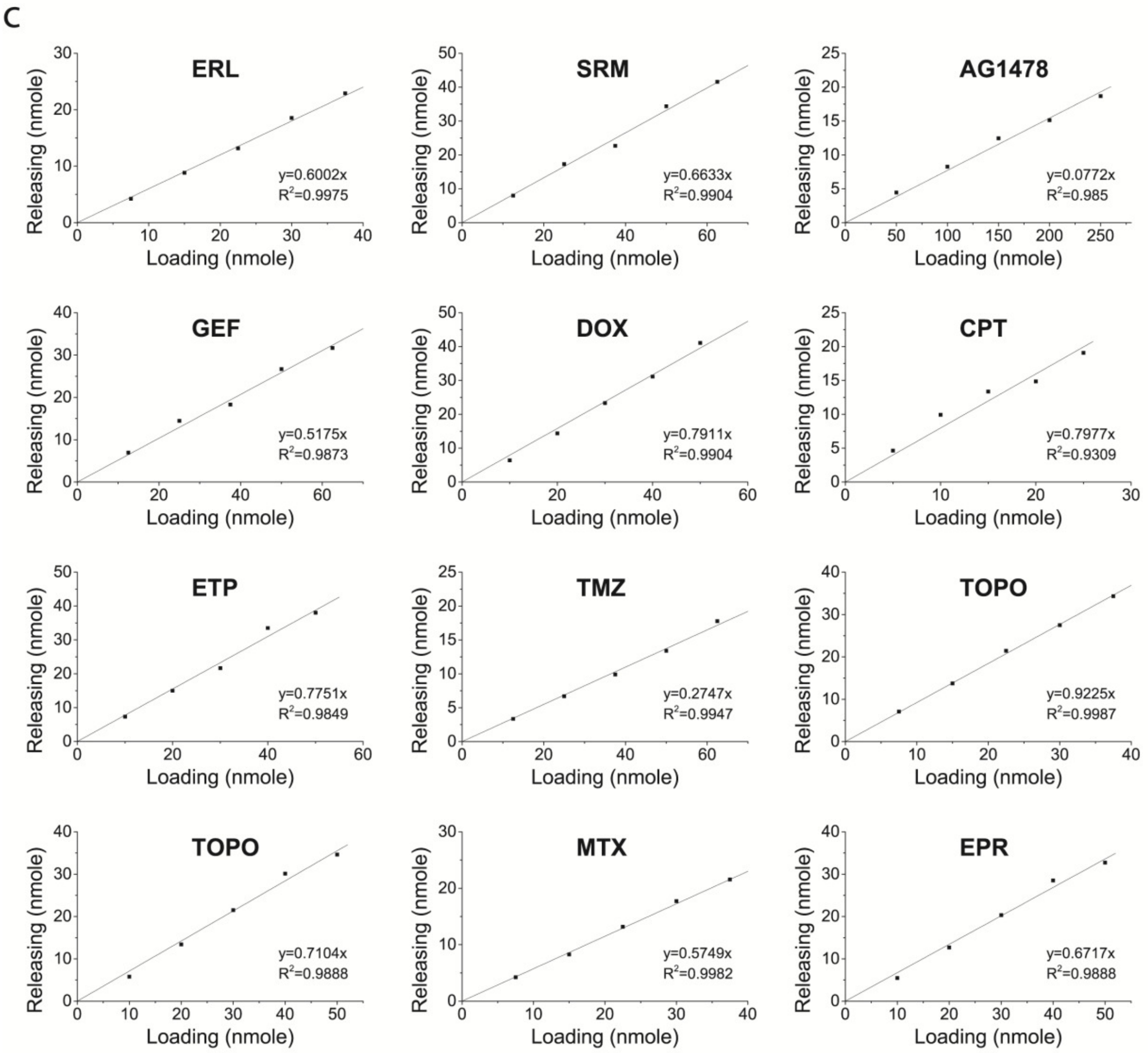

Figure S3. Loading-releasing relationship of all drugs. Schematic of the bulk-scale loading-releasing experiment is shown in Figure 2b. Because it is hard to measure released drug concentration in a single microwell on the cell chip due to its small volume, the releasing ratio was measured in a bulk-scale (See also Table S1, S2). a) Peak wavelength was determined from the absorbance spectrum of each drug. The absorbance spectrum was measured from UV-vis spectrophotometer (SHIMADZU, UV-1800) b) Reference curve of absorbance-concentration relationship. Peak wavelength is linear to the drug concentration. c) Released amount of drug is proportional to the initial loading amount. The releasing amount was calculated from the absorbance of the released solution. The slope of the graph represents releasing ratio.

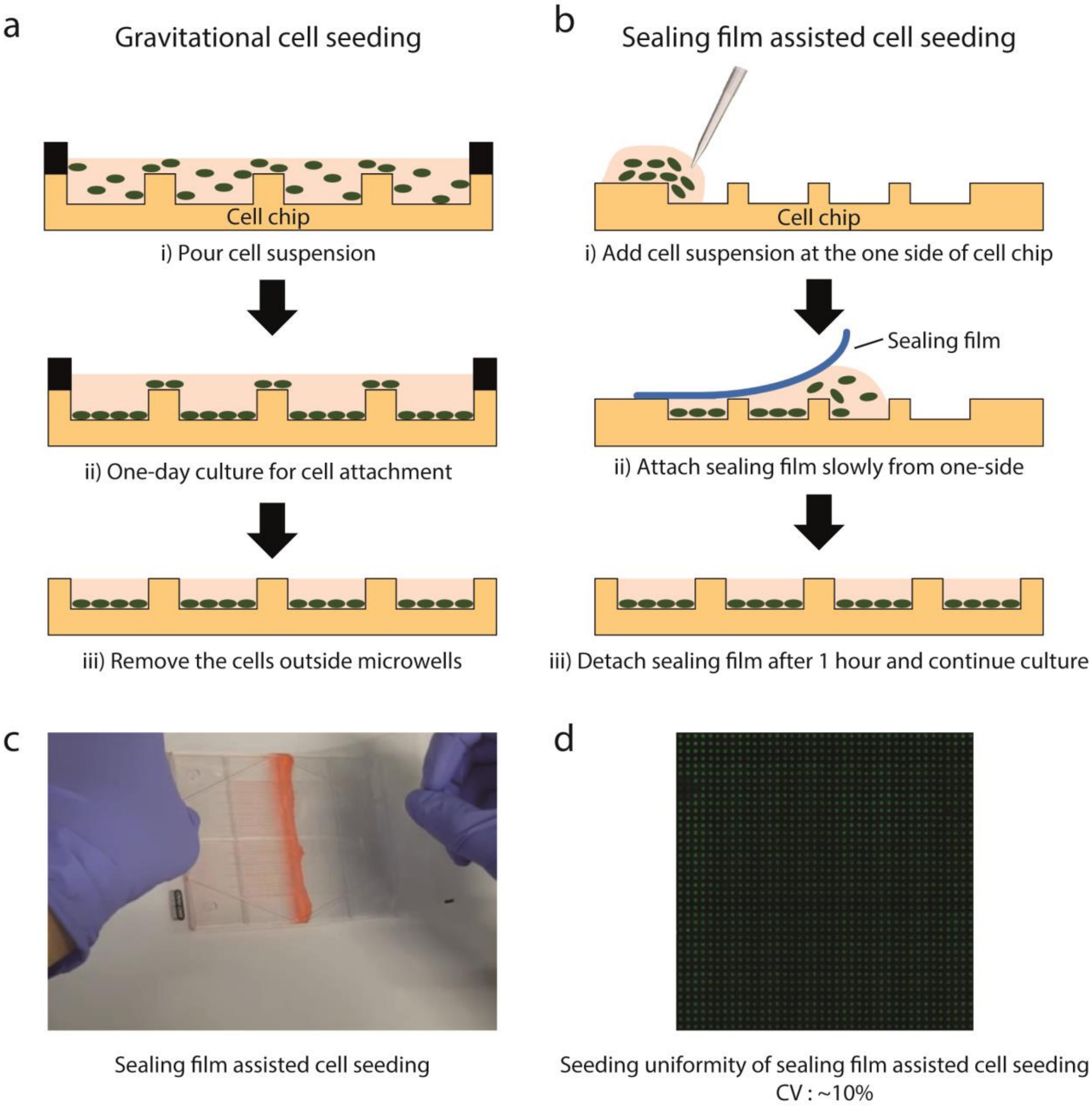

Figure S4. Process of cell seeding on the cell chip. a) Gravitational cell seeding. Pour the cell suspension on the cell chip and leave the cell chip in the incubator for one day to attach to the bottom of microwells. After one day, remove the cells attached out of microwells by using a scraper. b, c) Sealing film assisted cell seeding. Spray the cell suspension on one side of the cell chip and gently adhere the sealing film from the side. After one hour, the cells stick to the bottom, and then the sealing film is removed. 320 μL of cell suspension was used to fill 1,600 microwells without bubbles, of which total volume is 160 μL. Cell number per microwell was in average 104.7, and CV value was 8.7%. d) Calcein AM staining results of a whole chip on which cells were seeded using sealing film assisted method. The fluorescence intensity from each microwell was uniform (CV: ~10%).

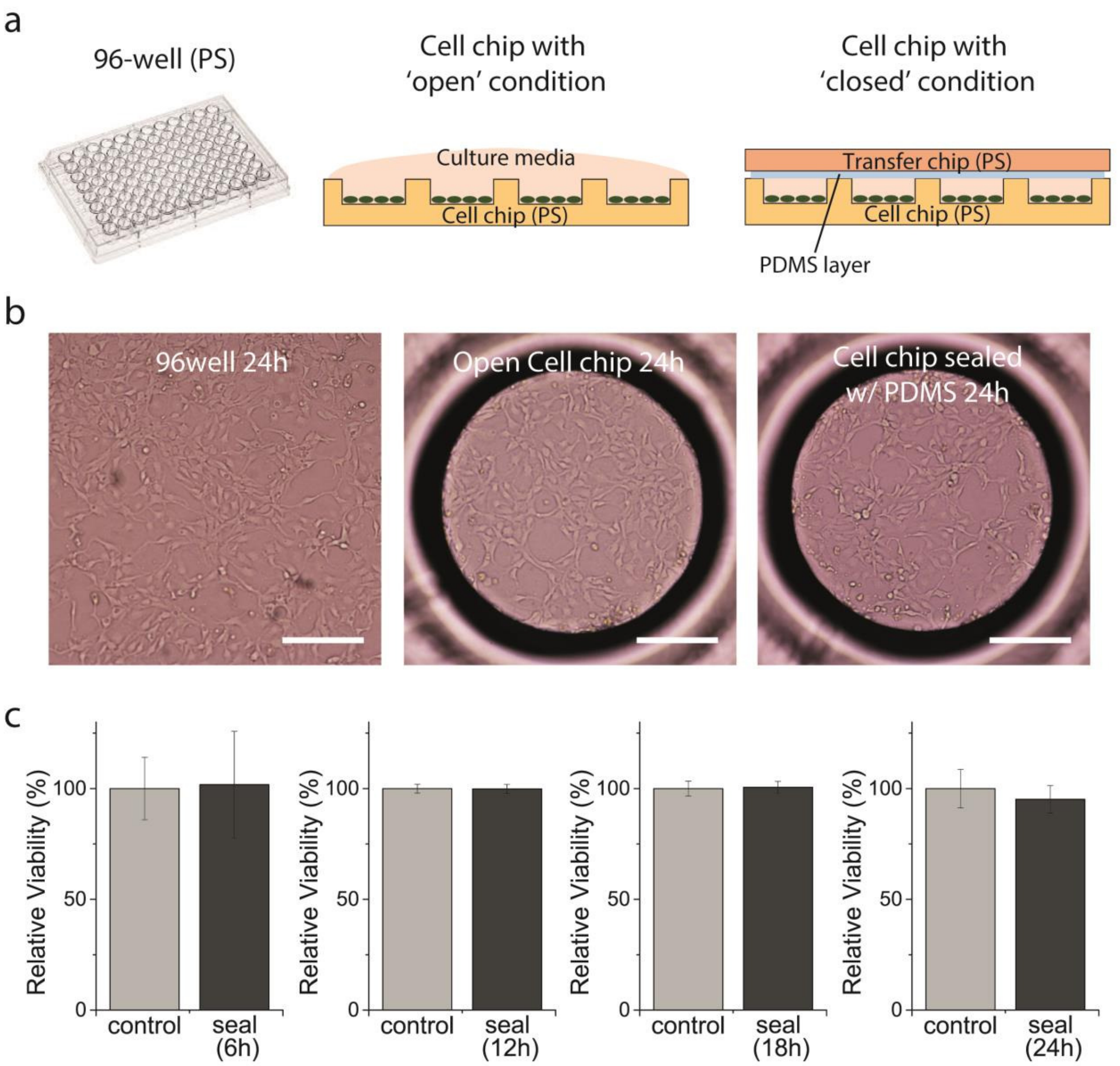

Figure S5. Effect of sealing the microwells during the incubation. a) Three experimental conditions were investigated. Cells cultured on a cell chip combined with transfer chip were compared with cells on the 96 well plate and the cell chip with 'open' condition, as a control. b) Morphology of cells after one day incubation. There was no significant morphological change due to the sealing. Scale bar: 200 µm. c) Viabilities of cells under 'open' and 'closed' condition were compared. For the 6, 12, 18, and 24 hour sealing, there was no decrease in the survival rate.

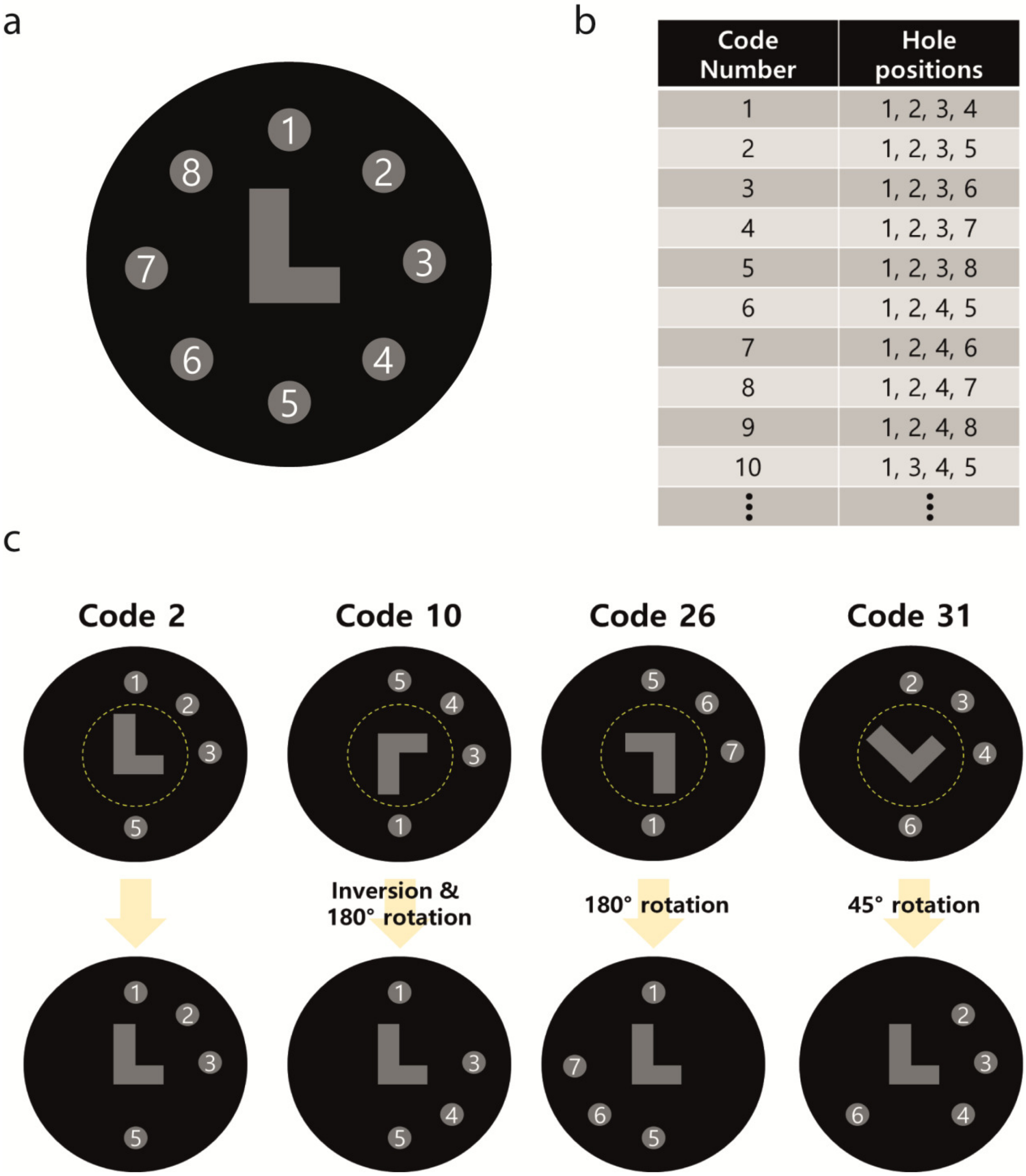

Code Number	Hole positions
1	1, 2, 3, 4
2	1, 2, 3, 5
3	1, 2, 3, 6
4	1, 2, 3, 7
5	1, 2, 3, 8
6	1, 2, 4, 5
7	1, 2, 4, 6
8	1, 2, 4, 7
9	1, 2, 4, 8
10	1, 3, 4, 5
⋮	⋮

Figure S6. Assigning code number according to the location of code circles. a) Among eight possible locations, four positions are occupied by code circles. b) The Code number of the microparticle is determined as shown in the table. c) Long code and short code are used to align the direction of a microparticle. Without these coding components, different codes cannot be distinguished since the location of code circles can be overlapped as a microparticle is rotated or inverted.

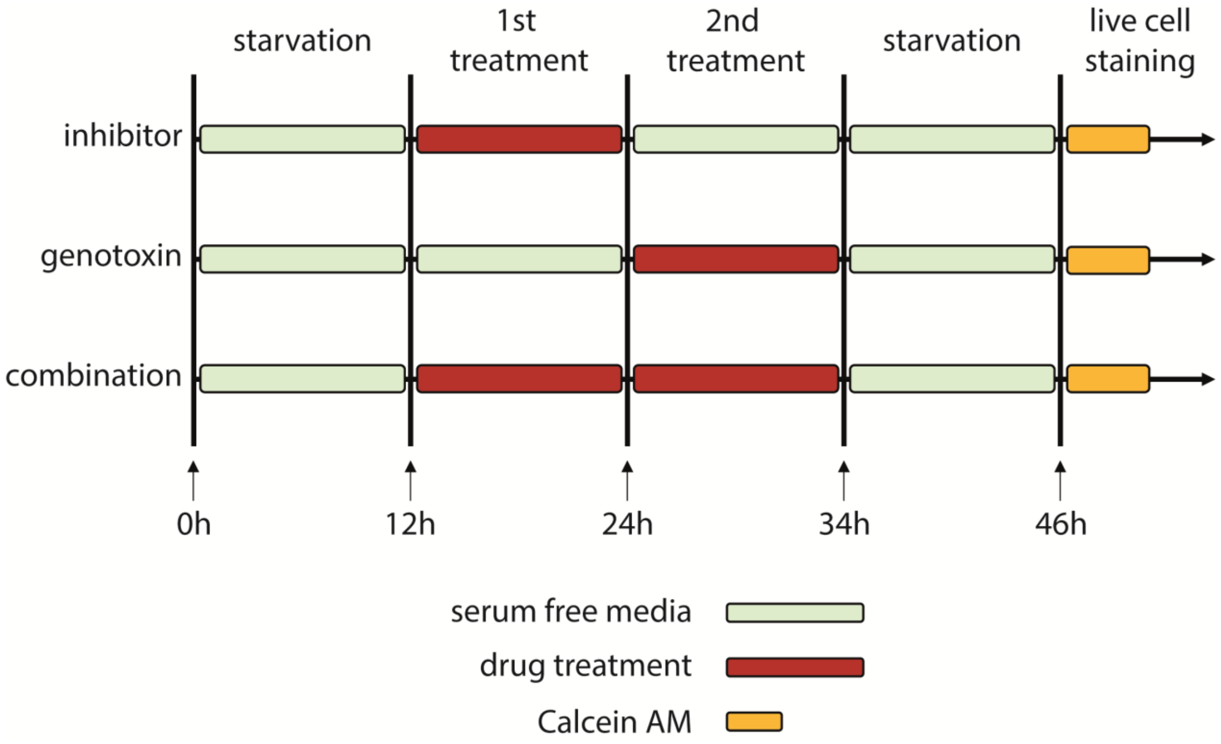

Figure S7. Time table of sequential combination cytotoxicity assay. After stable attachment of cells, the cells were starved under serum-free culture media for 12 h. Then, the first drug was applied for 12 h. Subsequently, previous culture media was washed out and the second drug was applied for 10 h. 12 h later, the viability of cells was measured using live cell staining with Calcein AM.

Supporting Tables

Name	Abbreviation	Code number	Type	Releasing ratio	Loading conc. to target 10μM
Erlotinib hydrochloride	ERL	1	EGFR inhibitor	60%	1.01 mM
Suramin sodium salt	SRM	2	Tyrosine kinase inhibitor (PDGF, EGF, etc.)	66%	0.905 mM
Tyrphostin AG 1478	AG1478	3	EGFR inhibitor	7.7%	7.76 mM
Gefitinib, free base	GEF	4	EGFR inhibitor	52%	1.16 mM
Doxorubicin hydrochloride	DOX	1	Genotoxin	79%	0.758 mM
(S)-(+)-Camptothecin	CPT	2	Genotoxin	80%	0.752 mM
Etoposide	ETP	3	Genotoxin	78%	0.778 mM
Temozolomide	TMZ	4	Genotoxin	27%	2.18 mM
Topotecan hydrochloride hydrate	TOPO	5	Genotoxin	92%	0.650 mM
Daunorubicin hydrochloride	DNR	6	Genotoxin	71%	0.845 mM
Mitoxantrone dihydrochloride	MTX	7	Genotoxin	57%	1.04 mM
Epirubicin hydrochloride	EPR	8	Genotoxin	67%	0.907 mM

Table S1. List of drugs used for sequential combination cytotoxicity assay. Releasing ratios were calculated from reference curves and loading-releasing curves in the Figure S3. Based on releasing ratio of each drug, required concentration of loading solution was calculated. 25 μL of loading solution dissolved in DMSO was used for drug loading into 15,225 microparticles.

Dimension of microwells on the Cell chip	
**Radius**	300 μm
**Depth**	350 μm
**volume**	99 nl

Number of microparticles to releasing volume ratio	
Number of microparticles	Volume (μl)
1	0.09891
15225	1500

Table S2. (Left) The dimension of microwells on the cell chip. Each microwell has 99nl of volume. (Right) Number of microparticles to releasing volume ratio. Drug molecules impregnated in one microparticle are released into one microwell (99 nL) on the cell chip when drug-releasing hydrogel microarray-on-a-chip is combined with cell chip. The ratio between single microparticle and releasing volume (99 nL) is equivalent to that of 15,225 microparticles to 1,500 μL of releasing volume. Because drug concentration of single microwell is difficult to measure due to the small volume, the bulk-scale loading-releasing experiment was performed to measure releasing ratio of each drug. In order to minimize the effect of volume ratio, 15,225 microparticles and 1,500 μL of releasing volume were used for that experiment. The experimental process and results are represented in Figure 2b and Figure S3c, respectively.

Number of microwells that can be tested with biopsy samples (5×10^5 cells)

	Partipetting	96-well
Volume of microwell (μl)	0.1	100
Cell number per microwell	105	1.0×10^4
Required cell number per microwell	200	1.0×10^4
Cell number in biopsy sample	5×10^5	
Number of microwell testable with biopsy sample	2500	50

Number of drug candidates that can be tested with biopsy sample

5×10^5 Cells	Partipetting	96-well (n=3)
$n \geq 3$	182	16
$n \geq 5$	144	
$n \geq 10$	100	

Table S3. Number of drug candidates that can be screened with a limited number of cells. Number of cells that can be used for screening is assumed as 5×10^5. (Left) Comparison between Partipetting and 96-well plate. With the decided number of cells, cell seeding onto 50 and 2,500 microwells are available for 96-well plate and Partipetting platform, respectively. (Right) Number of drug candidates to be screened guaranteeing duplications over n. For the Partipetting, number of screenable drug candidates were obtained from Monte Carlo simulation result that probability was over than 0.98.
